# Evaluating the efficiency of CHEF and CMV promoter with IRES and Furin/2A linker sequences for monoclonal antibody expression in CHO cells

**DOI:** 10.1371/journal.pone.0185967

**Published:** 2017-10-12

**Authors:** Saeedeh Ebadat, Samira Ahmadi, Maryam Ahmadi, Fatemeh Nematpour, Farzaneh Barkhordari, Reza Mahdian, Fatemeh Davami, Fereidoun Mahboudi

**Affiliations:** 1 Biotechnology Research Center, Pasteur Institute of Iran, Tehran, Iran; 2 Medical Biotechnology Department, Semnan University of Medical Sciences, Semnan, Iran; 3 Molecular Medicine Department, Biotechnology Research Center, Pasteur Institute of Iran, Tehran, Iran; Justus Liebig Universitat Giessen, GERMANY

## Abstract

In recent years, monoclonal antibodies (mAbs) have been developed as powerful therapeutic and diagnostic agents and Chinese hamster ovary (CHO) cells have emerged as the dominant host for the recombinant expression of these proteins. A critical step in recombinant expression is the utilization of strong promoters, such as the Chinese Hamster Elongation Factor-1α (CHEF-1) promoter. To compare the strengths of CHEF with cytomegalovirus (CMV) promoter for mAb expression in CHO cells, four bicistronic vectors bearing either internal ribosome entry site (IRES) or Furin/2A (F2A) sequences were designed. The efficiency of these promoters was evaluated by measuring level of expressed antibody in stable cell pools. Our results indicated that CHEF promoter-based expression of mAbs was 2.5 fold higher than CMV-based expression in F2A-mediated vectors. However, this difference was less significant in IRES-mediated mAb expressing cells. Studying the stability of the F2A expression system in the course of 18 weeks, we observed that the cells having CHEF promoter maintained their antibody expression at higher level than those transfected with CMV promoter. Further analyses showed that both IRES-mediated vectors, expressed mAbs with correct size, whereas in antibodies expressed via F2A system heterogeneity of light chains were detected due to incomplete furin cleavage. Our findings indicated that the CHEF promoter is a viable alternative to CMV promoter-based expression in F2A-mediated vectors by providing both higher expression and level of stability.

## Introduction

The emergence of monoclonal antibodies (mAbs) as an effective therapeutic agent has opened a new approach for the treatment of various diseases [1]. Today, the majority of approved therapeutic mAbs are produced in Chinese hamster ovary (CHO) cell lines [2]. The creation of stable high producer cell line is one of the most crucial factors to support high demand of market [3]. To improve the productivity of cell line, designing optimal expression vector plays a key role [4].

An essential component of an expression cassette is promoter which contributes to expression level and stability of transgenes expression [5]. A commonly used promoter for driving recombinant protein expression in mammalian cells is human cytomegalovirus (CMV) major immediate-early (MIE) [4, 6]. Although CMV gives high level of gene expression, there are several reports demonstrating the susceptibility of this promoter to silencing. DNA methylations of CMV which is caused by epigenetic events can lead to a decrease in the production of antibody [7, 8]. Alternatively, CHO-specific constitutive promoters are good candidates. CHO-derived elongation factor-1 promoter (CHEF-1) has been shown to be a successful system to obtain high level of expression in mammalian cells [9, 10].

Apart from designing an optimal expression cassette with the strong promoter, which is common for all recombinant proteins, there is another challenge for mAbs production that should be taken into account [6]. The most common types of mAbs in the market, immunoglobulin G (IgG), are comprised of two identical light chains (LC) and two identical heavy chains (HC) polypeptides. Therefore the balanced ratio of the light to heavy chains has a great effect on the optimal production of recombinant IgG in mammalian cells. Generally, the recombinant mAb expression is attained either by cotransfection of two separate vectors each encoding one chain of antibody or transfection of a single vector encoding both chains. Several studies have shown that employing a single vector provides a tighter control over the expression ratio of LC/HC [6, 11, 12]. Co-expression of heavy and light chains under the control of a single promoter can be achieved using either viral internal ribosome entry site (IRES) or viral 2A-self cleavage sequences [13].

When IRES element is introduced between two open reading frames (ORFs), the first ORF is translated via cap-dependent mechanism. The translation of second ORF downstream of IRES element is initiated through a cap-independent mechanism which is mediated through IRES sequences [14, 15]. However, IRES-driven cap-independent translation is less efficient than cap-dependent translation [16].

2A-self cleavage sequences present an alternative approach to express an equal amount of light and heavy chains from a single open reading frame. They are short, highly conserved sequences which are originally derived from the foot and mouth disease virus (FMDV). The insertion of 2A peptide between LC and HC genes leads to the generation of separate light and heavy chains via cotranslational cleavage [17, 18]. Upon the self-cleavage of 2A-sequences, parts of 2A amino acids remain attached to the upstream protein. To generate authentic polypeptide, furin cleavage site can be inserted before 2A sequences. After furin cleavage, the upstream protein contains two to four C-terminal basic amino acids (R or K) that can be removed by carboxy peptidases (CPs) [19].

In the present study, we have investigated the efficiency of CHEF and human CMV major immediate-early (MIE) promoters in the expression of mAb in stable CHO cell lines. To this purpose, we have constructed bicistronic vectors employing either IRES or 2A-peptide sequences. We further evaluated the effect of these linkers on the level of the mAb expression and compatibility of these linkers with CHEF promoter.

## Materials and methods

### Synthesis of full-length antibody

A two-step gene synthesis protocol was employed to link light and heavy chain of the IgG1 model antibody, mAb0014, with furin/2A sequences. This method relied on two separate PCRs and an overlap extension PCR. Using overlapping primers, the sequences encoding furin cleavage site (RRKR) and 2A peptide (APVKQTLNFDLLKLAGDVESNPGP), derived from a foot and mouth disease virus (FMDV), were inserted between the light and heavy chain. These processes in detail were as following:

#### Primer design

To generate full-length antibody, two pairs of primers were designed. Primer design was the crucial step for the success of this procedure. These primers which allowed insertion of furin/2A peptide between two chains of mAb consisted of two outermost primers (LC-forward and HC-reverse) and two chimeric primers (LC-reverse and HC-forward). Outermost primers contained restriction enzyme sites at their 5' ends, and chimeric primers comprised of two major parts, the complementary region that was derived from the sequence (furin/2A) to be inserted and the template-annealing region.

Based on the native amino acid sequences coding furin and 2A peptide (28 a.a.), the codons of these genes were optimized by employing the codons more frequently used in CHO. To keep GC content within 40–60% and avoid the formation of secondary structure and dimer primers, high-frequency codons were not always chosen, whenever the differences between the codon frequencies were not significant. Primer designing was carried out using the oligo6 software. The sequences of all oligonucleotide sets are provided in Table 1.

**Table 1:** Primers used for construction of the LC-furin-2A-HC gene.

Primer Name	Sequences(5′-3′)	Restriction Sites
**LC Forward**	CAG**GAATTC** GCCGCCACCATGTACC **(25 nt)**	EcoRI
**LC Reverse**	CAGCAGGTCGAAGTTCAGGGTCTGCTTCACGGGGGCCCTCTTTCTCCTACATTCGCCACGGTTGAAAC **(68 nt)**	
**HC Forward**	ACCCTGAACTTCGACCTGCTGAAGCTCGCCGGAGACGTGGAGTCCAACCCCGGCCCCATGTATCGTATGCAGCTGCTGTC **(80 nt)**	
**HC Reverse**	TGC**TCTAGA**TTATCATTTACCAGGGGACAG **(30nt)**	XbaI

#### PCR amplification of heavy and light chains

DNA sequences encoding light and heavy chains were formerly synthesized on two separate vectors (GenScript, US) [20] and were simultaneously amplified by two different PCRs. Each PCR reaction was performed in 25μl mixture containing ddH_2_O, PCR buffer, dNTPs (0.2mM), MgCl_2_ (2.5 mM), forward primer (2.5 mM), reverse primer (2.5 mM), 2.5 U PWO high-fidelity DNA polymerase (Roche, Germany), and 50 ng of plasmid DNA carrying either light or heavy gene as a template. All amplifications were carried out with a Master Cycler Gradient Thermocycler (Eppendorf, Germany). Synthesis of light chain was conducted under the following program; initial denaturation at 94°C for 3 min, followed by 35 cycles of denaturation at 95°C for 1 min, annealing at 67°C for 40 sec, extension at 72°C for 1 min, and a final extension of 10 min at 72°C. Heavy chain PCR profile was: 3 min at 94°C, continued for 1 min at 95°C, 45 sec at 57.5°C, 1 min at 72°C which was repeated for 35 cycles, and 10 min at 72°C. All amplified fragments were electrophoresed in 1% agarose gel.

#### Overlap extension PCR

PCR products from the pair of first PCR reactions were directly used as templates to assemble into full-length antibody by overlap extension PCR. 1μl of each template was subjected to PCR amplification in the presence of 25 μl PCR mix composed of ddH2O, PCR buffer, 2.5mM MgCl_2_ and 0.2mM dNTP, 2.5 mM of two outer most primers (LC- forward and HC- reverse), and 5 U long enzyme PCR (Fermentase, Thermo Fisher Scientific, US). Assembly reaction was performed under the following conditions: 3 min initial denaturation at 95°C, 35 cycles of 95°C for 1 min, 58°C for 45 sec, and 68°C for 1 min, and a final extension at 68°C for 10 min.

### Vector construction

**Fig 1:**
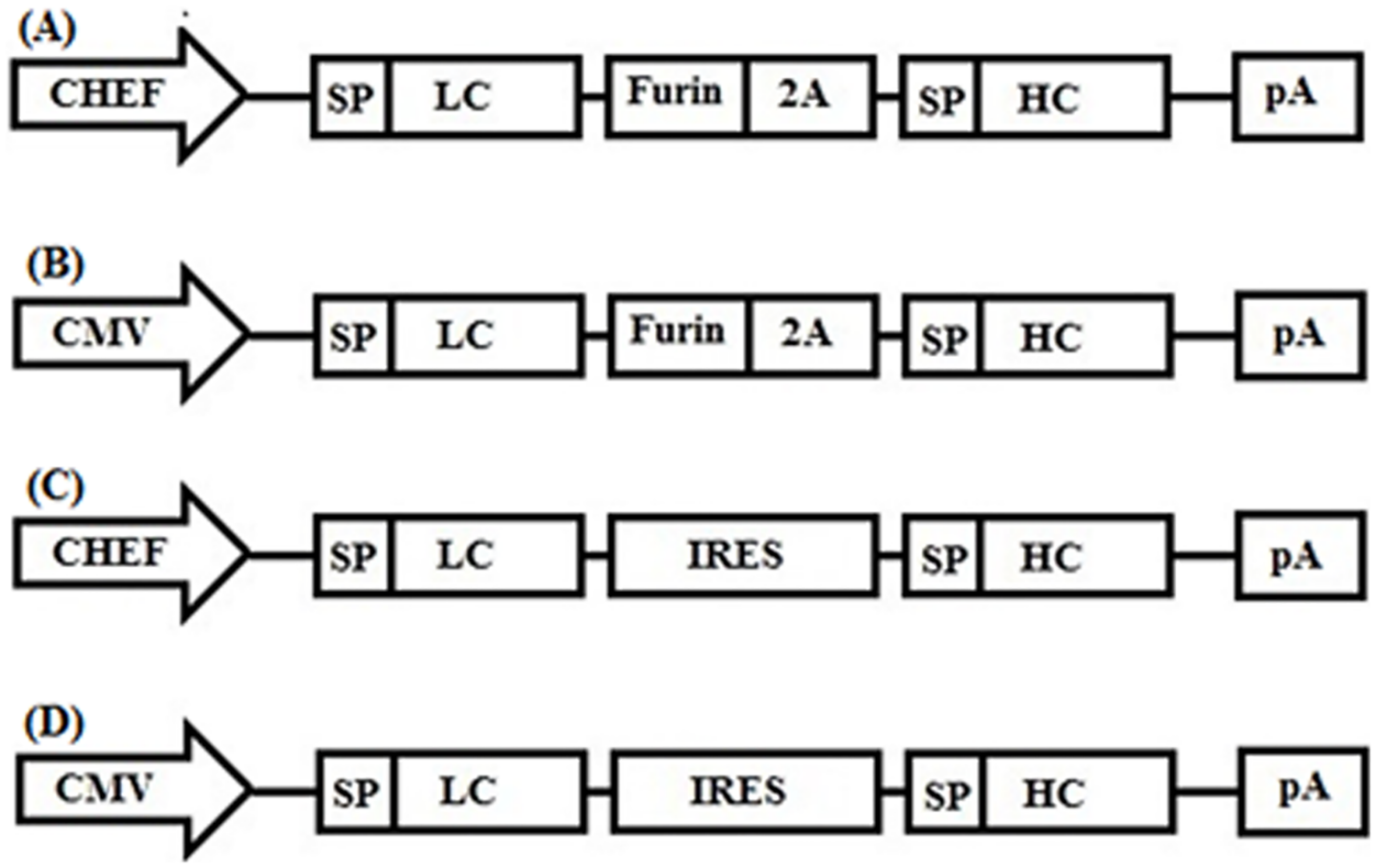
Schematic representation of the vectors containing different promoters and furin/2A and IRES sequence for mAb expression. (A) CHEF-LC-F2A-HC (CHEF-F2A) vector, (B) CMV-LC-F2A-HC vector, (C) CHEF-LC-IRES-HC vector, (D) CMV-LC-IRES-HC vector. CHEF; CHEF promoter, CMV;CMV promoter, SP; signal peptide, LC; Light chain, Furin; Furin recognition site sequence, 2A; 2A self-cleavage sequence derived foot-and-mouth disease virus (FMDV), IRES; Internal ribosome entry site, HC; Heavy sequence, pA; poly A.

Four different bicistronic constructs expressing the full-length antibody were created: CMV-LC-Furin-2A-HC (CMV-F2A) and CMV-LC-IRES-HC (CMV-IRES) which contained CMV promoter and CHEF-LC-furin-2A-HC (CHEF-F2A) and CHEF-LC-IRES-HC (CHEF-IRES) expressing the whole antibody under the control of CHEF promoter. In all constructs, the light chain was located upstream of the heavy chain, and signal sequences for both chains were included to ensure proper secretion of the resultant full-length antibody (Fig 1). The PB513b1 vector (System Biosciences (SBI), US) was utilized as a backbone vector. All vectors were constructed based on cloning methods [21]. The construct CMV-F2A was prepared as following: initially, the newly generated PCR product which contained LC-Furin-2A-HC sequences with 3'A overhang was gel purified and subjected to direct cloning in T/A vector, ptz57RT (Thermo Fisher Scientific, US). Subsequently, antibody fragment was recovered from ptz57R/T vector using ECoRI and XbaI, subcloned into PB513b1 vector digested with ECoRI and XbaI, respectively. Plasmid bearing LC-IRES-HC sequences was previously constructed in our lab [22]. LC-IRES-HC fragment was excised with NheI and NotI and inserted in PB513b1 vector to create CMV-IRES vector [23]. Promoter region (positions –1473 to –16 to the initiating ATG) derived from CHEF-1 gene (Genbank accession number AY188393) was synthesized by GeneScript [9, 10]. To generate the vectors employing CHEF promoter for expression of the antibody, CMV sequence of CMV-F2A and CMV-IRES vectors was cut by SpeI and NheI digestion and substituted by sequences coding CHEF promoter. The accuracy of all recombinant vectors was validated by restriction analysis and DNA sequencing.

### Cell culture

Free Style^™^ CHO-S cells (Gibco, Life Technology, USA) were cultured in suspension using proCHO5^™^ medium (Lonza AG, Verviers, Belgium) supplemented with 4mM L-glutamine (Gibco, Life Technology, US), anticlumping, pluronic acid, and Pen-Strep (Gibco, Life Technology, US). Cells were maintained in T-Flak (Greiner, Belgium) or shaken in cylindrical glass bottles and incubated at 37°C in a 5% CO_2_ incubator. CHO-S cells were routinely subcultured every 3–4 days, and at each passage, 5*10^5^ cells per milliliter were seeded. Cell density and viability were determined by trypan blue using a hemacytometer.

### Transfection

CHO-S cells were transfected with constructed vectors using X-tremeGENE HP DNA transfection reagent (Roche, Germany). Prior to transfection, plasmids were purified, using plasmid mini-kit (Roche, Germany) and 7.5*10^6^ cell/well with 90% confluency were seeded in a 6-well plate (Greiner, Belgium). Plasmid DNA was complexed with DNA transfection reagent at a ratio of 1:3 according to the manufacturer’s instructions. The mixture of 3 μg plasmid DNA and 9 μl transfection reagent in the final volume of 300μl serum free media (SFM) was prepared. After 30 minutes incubation at room temperature, the complex solution was added drop-wise to each well. Since green fluorescent protein (GFP) was co-expressed on the same expression vectors, transfection efficiency in transfected cell population was analyzed by flow cytometry method. All transfections were carried out in triplicate.

### Development of stable CHO-S cell lines expressing mAb

Generation of stable CHO cell pools expressing mAb was achieved by introduction of four expression vectors. CHO-S cells were transfected with recombinant vectors as described earlier. Forty eight hours after transfection, an antibiotic selection started by adding medium containing selection marker to the cells. The media was removed and replaced with proCHO5^™^ medium containing 10μg/ml puromycin (Sigma, USA). The optimum concentration of puromycin was determined based on minimum inhibitory concentration (MIC) assay. Cells were routinely passaged with selection media. When the cell viability recovered above 80%, they were transferred from the 6-well plate to T-25 flask. Cells were cultured for 25 more days with continuous puromycin selection until a pool of puromycin resistant stable cells expressing mAb was selected. Culture supernatants of stable cell pools were analyzed by ELISA.

### Stability studies

To evaluate the long-term stability of mAb expression, the cell pools were maintained in suspension culture without any antibiotic pressure. Approximately, every 14 days, expressed mAb was screened by ELISA. This study was performed for 18 weeks.

### Clonal selection

Single cell clones were obtained by limiting dilution technique. Cells from stable cell pools were seeded in 96 well plates at a density of 0.5–1 cell per well. Cells were kept for 21 to 28 days, till individual colonies were formed. The positive clones were picked up and cultured separately in 24 well plates and subsequently expanded in a 6-well plate. After 7 days, the cell culture supernatants were collected, and mAb concentration was measured by ELISA.

### ELISA assay

The antibody titer in cell supernatants was measured using a sandwich-ELISA assay. The 96-well plates were coated with rabbit anti-human IgG-Fc gamma secondary (Pierce, Thermo Fisher Scientific, US) which was diluted 1:16000 in carbonate/bicarbonate buffer, pH 9.6. After an overnight incubation at 4°C, the plates were washed twice with PBS containing 0.05% tween-20 (PBST) and then blocked with 150 μl of BSA 0.5% (Roche, Germany) for one hour at 37°C. Following three washes with PBST, the plates were coated with 100 μl cells supernatants for one hour at 37°C. After three-step washes, horseradish peroxidase-conjugated goat anti-human IgG (Sigma, Germany) was diluted 1:20,000 in PBS, and 100 μl per well was added. The plates were incubated at 37°C for one hour. After washing the plates with PBST for three times, 100 μl 3,3’,5,5’-tetramethylbenzidine (TMB) (Sigma, USA) was added and developed at room temperature for fifteen minutes. The reaction was stopped by adding 100 μl H_2_SO_4_ (2N). The reaction absorbance was read at 450 nm, and protein concentration was estimated based on the human IgG1 standard curve.

### Purification

The supernatant of CHO-S stable cell pools expressing antibody was collected after 10 days. The supernatant was harvested by centrifuging at 7000 rpm for 30 min followed by filtration with 0.22 membranes. Supernatant containing antibody was purified using Protein-A column (GE Healthcare, Sweden). Following the column equilibration with PBS Buffer, the sample was directly loaded on the column. Column loading was controlled by measuring the absorbance at 280nm. After sample loading, the column was washed with equilibration buffer. Then, the bound antibody was eluted with 0.1 M sodium citrate buffer, pH 3. Eluted fractions were collected and neutralized with 2M Tris-HCl, pH 8. They were analyzed by SDS-PAGE and western- blotting.

### SDS- PAGE and western blotting

Purified antibodies were subjected to electrophoresis on 12% SDS-PAGE gels using a Mini-PROTEAN II apparatus (Bio-Rad, USA). Twenty five microliters of each fraction were denatured and reduced in SDS sample buffer and finally loaded on the gel. The gel was stained with coomassie brilliant-blue. Following separation of samples on SDS-PAGE, western blot was performed. Samples were transferred to nitrocellulose membrane employing a semi-dry system (BioRad, USA). After an overnight incubation of the membrane with skim milk 5%, the membrane was washed three times with PBST. Later, it was incubated with 1:1000 diluted horseradish peroxidase-conjugated goat anti-human IgG (Sigma, Germany) for two hours and then washed three times with PBST. Finally, the color was developed in 3,3’-diaminobenzidine tetrahydrochloride (DAB) (Sigma, Germany) for 3–5 min. Relative intensities of the protein bands for each lane were quantified using the Quantity One software (BioRad). Furin cleavage efficiency was calculated based on the following formula: cleavage efficiency = cleaved form/(cleaved form+ uncleaved form) [24].

### Quantification of transgene expression by real-time PCR

The relative expression level of mAb mRNA in transfected cell lines was quantified using Quantitative Real Time PCR (qRT-PCR). Total RNA was extracted from 1*10^6^ cells using TRIzol reagent (Sigma, US). RNA concentration and purity was measured by its absorbance at 260 and 280 nm. To remove potential contamination of genomic DNA, one microgram of isolated RNA was treated with DNase I (Fermentas, Thermo Fisher Scientific, US). First strand complimentary DNA, cDNA, was prepared using cDNA synthesis kit (Roche, Germany) according to the manufacturer’s instruction. For each reaction, 1μg RNA /10 μl of final volume was used. cDNA was diluted 1:5 in RNase-free water, and qRT-PCR reactions were performed using Sybr-Green PCR Master Mix (Applied Biosystems, USA) in a final volume of 10 μl. Primer design was performed using the Primer3 software. The respective primers are listed in Table 2. Glyceraldehyde-phosphate dehydrogenase (GAPDH) was used as a reference gene. The qRT-PCR assays were performed on the Applied Biosystems 7500 Real Time PCR system (Applied Biosystems, USA), using the following cycling parameters: initial 10 min activation step at 95°C followed by 40 cycles of amplification, 15 sec denaturation at 95°C, 40 sec annealing at 65°C, and 35 sec extension at 60°C. All reactions were performed in triplicate. Amplification efficiencies were calculated by making a serial dilution of the target template and determining the CT value for each dilution. Data collected from qRT-PCR detection system were analyzed using relative quantification methods [25].

**Table 2:** Oligonucleotides used for analysis of mRNA levels and relative gene copy numbers by quantitative real-time PCR.

Target gene	Forward primers (5ʹ-3ʹ)	Reverse primers (5ʹ-3ʹ)
**Light Chain**	CAGAGTGTGGACTACGATGGAGAC	CGGAGCCTGAGAACCTGGATG
**β- Actin**	GAAGTGTGACGTCGACATCCGCAAAGAC	GGTTGACCTGGAAGGGCCCATCATG
GAPDH*	CACTCTTCCACCTTTGATGCTG	GTCCACCACTCTGTTGCTGTAGC

*GAPDH, glyceraldehyde-phosphate dehydrogenase

### Recombinant gene copy numbers quantification

Genomic DNA was extracted from 1*10^6^ cells and purified using the DNA isolation kit (Roche). The quality and concentration of DNA were determined measuring absorbance at 260/280 nm. Gene copy number (GCN) of antibody based on the light chain gene was quantified by qRT-PCR. 20 ng genomic DNA of each cell pool was used per reaction. Oligonucleotide sequences are summarized in Table 2. The qRT-PCR run was carried out as following: stage 1, 95°C for 10 min; stage 2, 95°C for 15 sec, 65°C for 40 sec, and 60°C for 35 sec which was repeated 40 times. Each run was conducted in triplicate. Raw data were analyzed as described by Osterlehner et al. [26].

## Results

### Insertion of furin recognition site and 2A sequences

**Fig 2:**
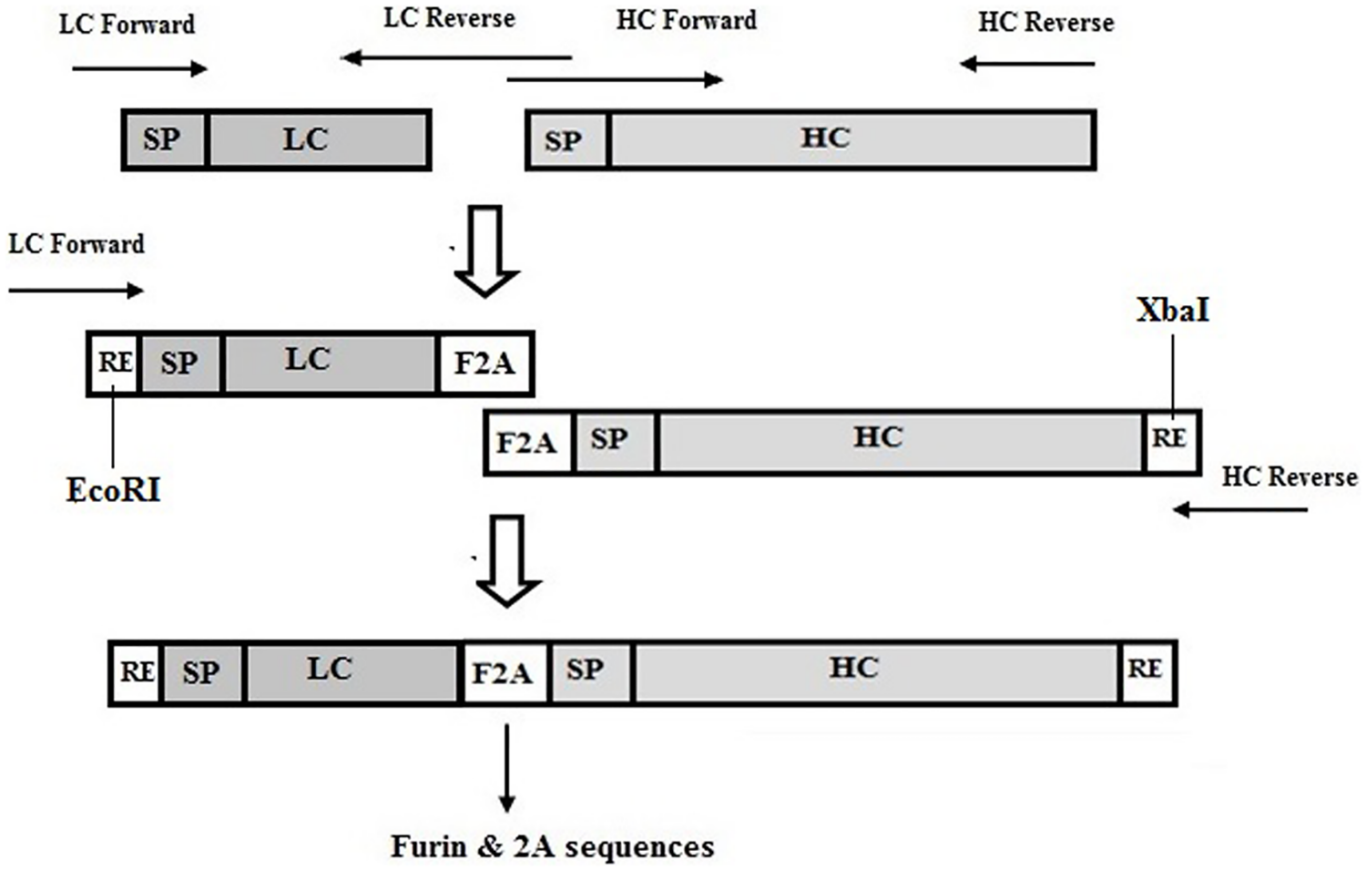
Schematic illustration showing insertion of furin/2A sequences between light and heavy chain by overlap extension PCR.

Generation of the fused PCR product, LC-Furin-2A-HC fragment, through overlap extension PCR is schematically shown in Fig 2. Light and heavy chain genes were amplified by two separate PCR reactions. Desired bands were observed at the positions of 780 and 1500 base pairs (bp) related to light and heavy chains. Overlapping sequences down-stream of the light chain and upstream of the heavy chain served as primers for the second PCR steps and contained furin/2A sequences. In the second round of PCR, both fragments were joined to form LC-Furin-2A-HC fragment. EcoRI and XbaI restriction enzyme sites were introduced in the upstream of the light and downstream of the heavy chain, respectively, which were used for insertion into the desired vectors.

### Development and evaluation of mAb expressing cell pools

**Fig 3:**
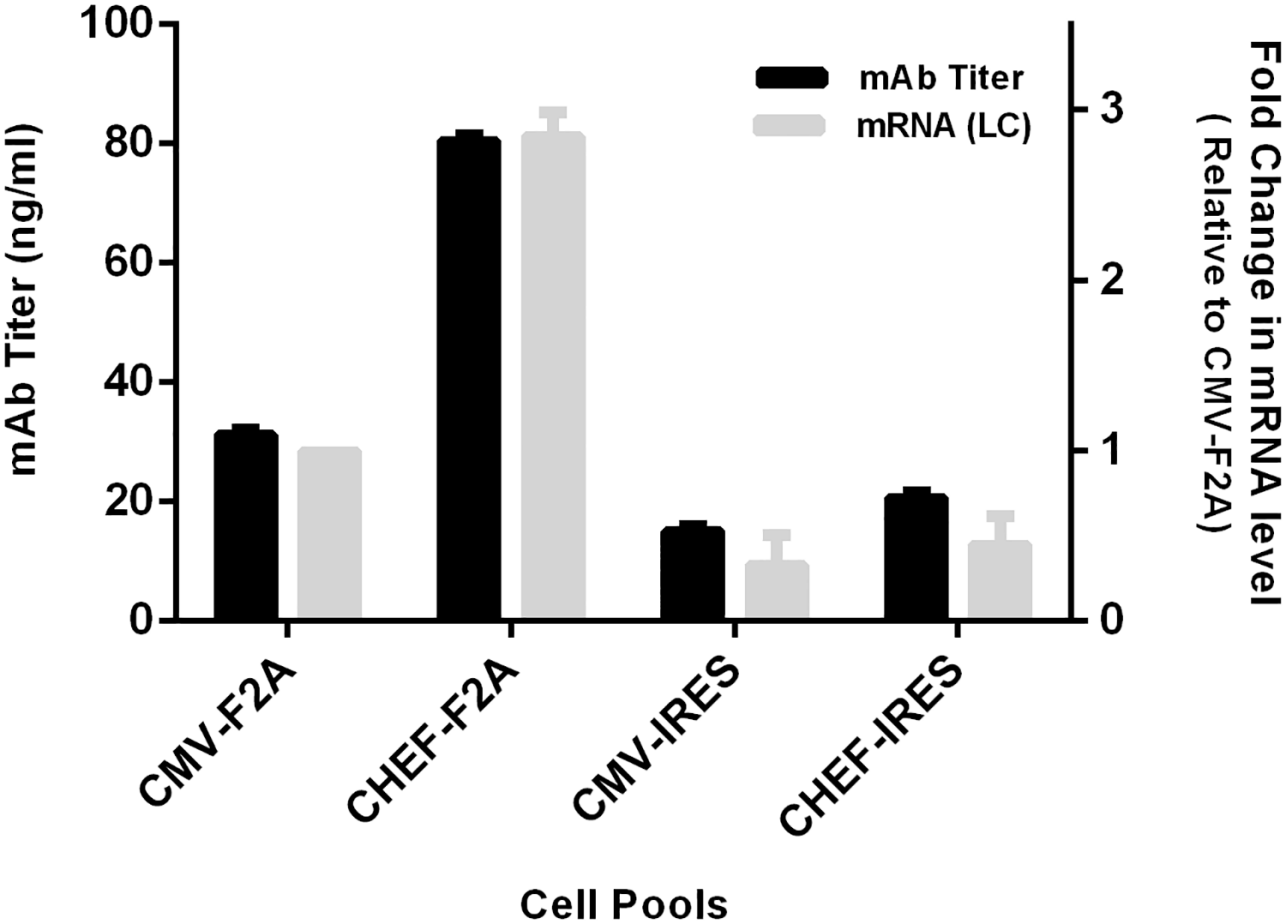
Analysis of the four bicistronic vectors for mAb and mRNA expression in established stable cell lines. Stably transfected pools were generated by transfection of CHO-S cells with various bicistronic vectors containing either IRES or F2A sequences and different promoters (CHEF or CMV). Levels of mAb and mRNA expression were measured by ELISA and qRT-PCR respectively. Black bars represent the mAb titer and gray bars represent mRNA fold induction. The error bars represent the standard deviation of three independent measurements.

Stable CHO-S cell lines expressing monoclonal antibody were generated by transfection of four different vectors. 48 hours post-transfection, cells showed viability above 80% and 25–30% of the cells expressed GFP. Stable cell pools were generated by culturing cells in puromycin containing media, and the selection process continued for a month. Measuring antibody titers in cell supernatants by ELISA assay indicated that CHEF promoter elicited higher expression in comparison to CMV promoter in both 2A and IRES expression systems although this difference was more noticeable in cell pools containing 2A-self cleavage sequences. In F2A system, the CHEF promoter enhanced antibody expression 2.5–2.8 fold more than CMV promoter while in IRES-systems minor differences were detected between two promoters. Considering the effect of both promoters and linkers, CHEF-F2A construct had the highest expression followed by CMV-F2A, CHEF-IRES, and CMV-IRES (Fig 3).

To investigate the correlation between mAb expression and its mRNA levels in four generated cell pools, qRT-PCR was performed. qRT-PCR analysis (Fig 3) confirmed the ELISA data and indicated that cells transfected with CHEF-F2A vector exhibited the highest expression in both the mRNA and protein levels.

### Purification and western-blot analysis of expressed mAbs

**Fig 4:**
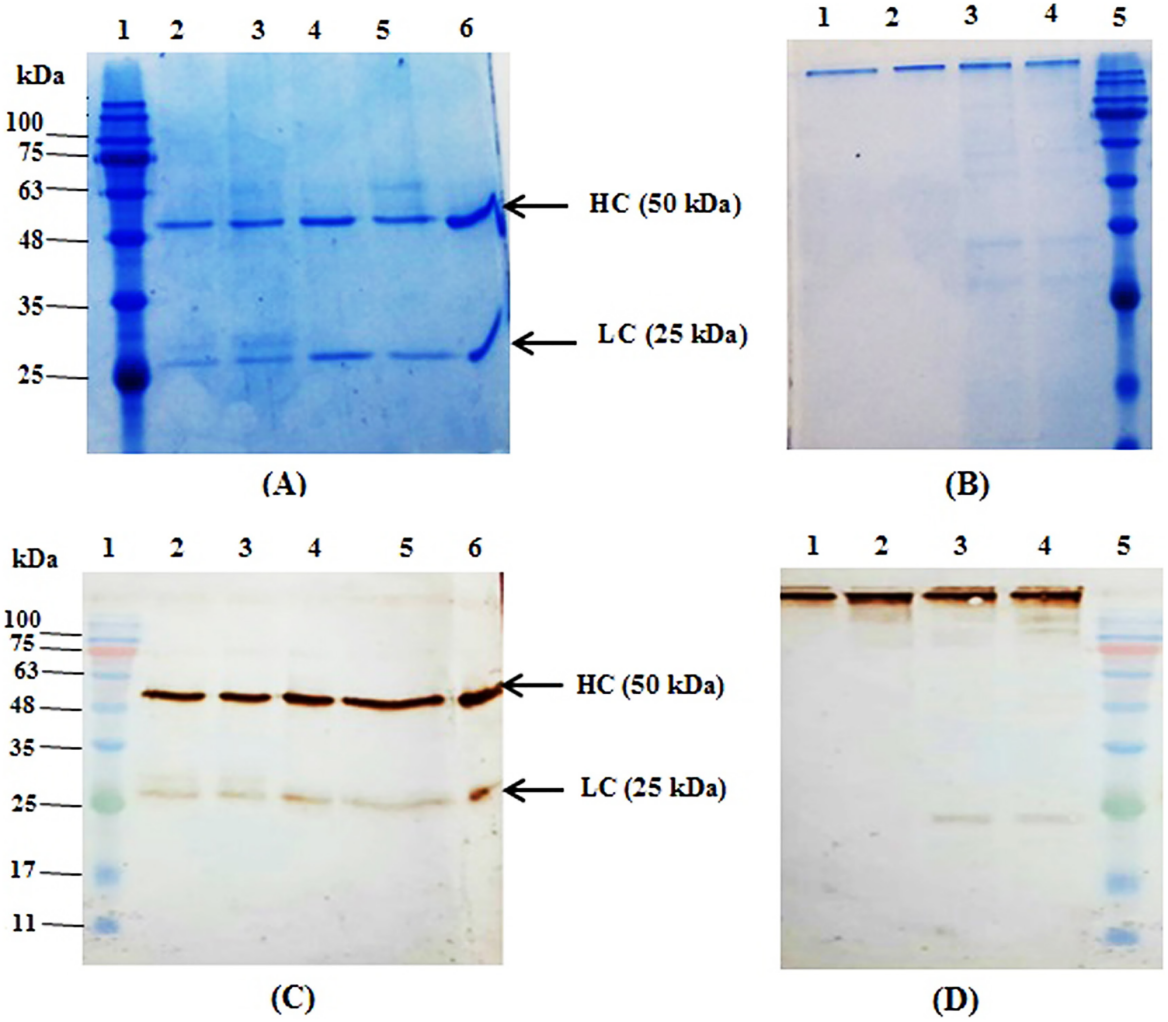
SDS-PAGE and Western blot analysis of purified mAb in stable cell pools transfected with four bicistronic vectors. Supernatants of three different cell pools were purified using protein-A. Purified samples were analyzed with SDS-PAGE and western blot under reducing and non-reducing condition. Commercial IgG1 was used as a positive control. (A) SDS-PAGE profile of purified samples in reduced state, lane 1; Protein molecular ladder, 2; CHEF-F2A, 3; CMV-F2A, 4; CHEF-IRES, 5; CMV-IRES, 6; positive control. (B) SDS-PAGE profile of purified samples in non- reduced state, lane 1; CMV-IRES, 2; CHEF-IRES, 3; CMV-F2A, 4; CHEF-F2A, 5; protein molecular ladder. (C) Western blot analysis of samples under reducing condition, lane 1; protein molecular ladder, 2; CHEF-F2A, 3; CMV-F2A, 4; CHEF-IRES, 5; CMV-IRES, 6; positive control. (D) Western blot analysis of purified samples in non- reduced state, lane 1; CMV-IRES, 2; CHEF-IRES, 3; CMV-F2A, 4; CHEF-F2A, 5; protein molecular ladder.

The monoclonal antibody was purified from the supernatant of cell culture using protein-A affinity chromatography. The purified products were separated by 12% commassie brilliant blue-stained SDS-PAGE, a 150 kDa band in non-reducing condition, and two bands of 50 kDa and 25kDa were observed in the reducing condition (Fig 4). The purified mAb was also evaluated by western-blotting (Fig 4). The detected bands in western blot were consistent with those detected on SDS-PAGE.

**Fig 5:**
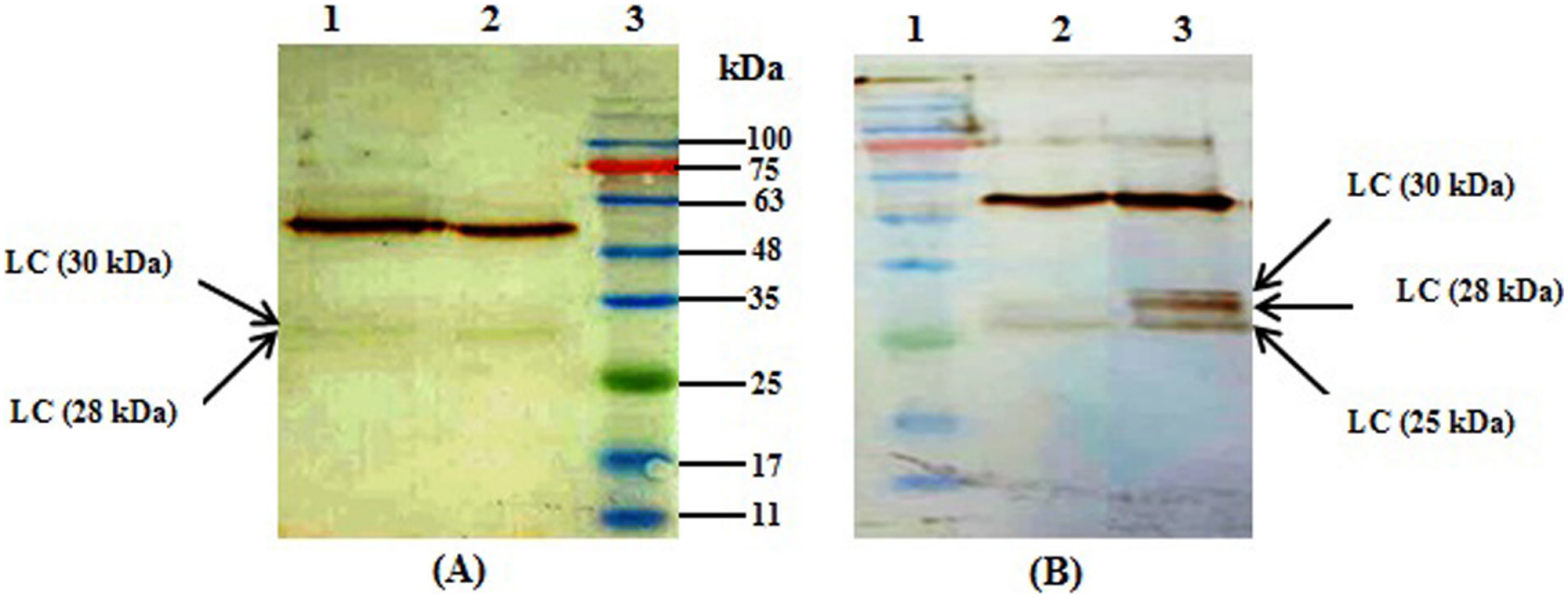
Western blot analyses of purified mAb in triplicate stable cell pools transfected with vectors bearing F2A-mediated expression system. Light chains with various molecular weights (25, 28 & 30 kDa) were detected. Triplicates of each stable pool were shown with 1, 2 and 3. (A) Lane 1; CHEF-F2A (2), lane 2; CHEF-F2A (3), lane 3; protein molecular ladder. (B) Lane 1; protein molecular ladder, lane 2; CMV-F2A (1), lane 3; CMV-F2A (3).

The antibodies expressed by F2A-mediated vectors were further analyzed under reducing conditions by western-blot. Interestingly, product patterns were not similar in all triplicate cell pools. Light chains with various molecular weights were detected (Fig 5). The expressed antibody had three bands at approximately 25, 28, and 30 kDa, corresponding to the light chains regardless of the promoter type. Complete cleavages at both 2A and furin recognition site would result in a 25 kDa light chain. Presence of bands at about 28 and 30 kDa could be explained by incomplete cleavage of furin or incorporated HC signal peptide, respectively. Lower intensity of the 30 kDa band in comparison to the other two bands and the absence of a higher molecular weight band corresponding to the fusion protein, LC-Furin-2A-HC, implies that 2A peptides cleaved more efficiently than furin. Relative intensities of these bands are demonstrated in Table 3.

**Table 3:** Analysis of furin cleavage efficiency in triplicate stable cell pools transfected with either CHEF-F2A or CMV-F2A vector. Cleavage efficiency was calculated by estimating density of cleaved form/ (cleaved form+ uncleaved form).

Cell Pools	25(kDa)	28(kDa)	30 (kDa)	Furin cleavage efficiency
**CMV-F2A (1)**	90%	5.5%	4%	90%
**CMV-F2A (2)**	75%	14%	11%	75%
**CMV-F2A (3)**	8%	81%	10%	8%
**CHEF-F2A (1)**	95%	3.5%	1.5%	95%
**CHEF-F2A (2)**	—	100%	—	0%
**CHEF-F2A (3)**	—	96.5%	3.5%	0%

### Evaluating the expression stability of cell pools generated by F2A system

**Fig 6:**
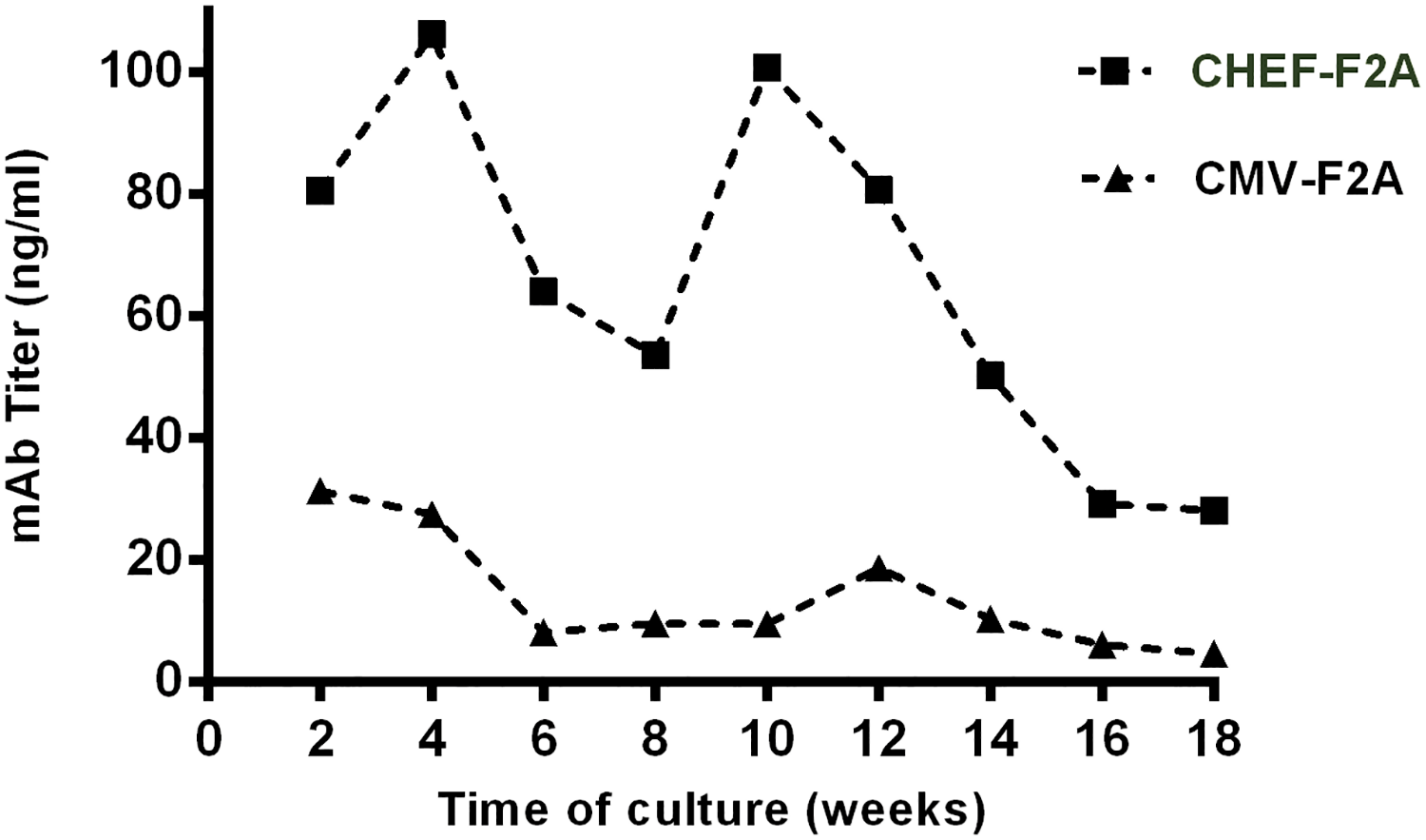
Analysis of the stability of antibody expression over time by stable cell pools transfected with two vectors containing F2A sequences. Both stable cells were cultivated for 18 weeks upon removal of puromycin as a selection marker. Every 2 weeks, antibody expression was monitored and measured by ELISA. The other pools exhibited the same expression pattern; the data from one of them was represented.

Since the long-term stability of the transgene expression is one of the major criteria in the development of stable cell line, the production stability was evaluated over several population doublings. To determine the impact of different promoters on the stability of mAb expression, cell pools with higher expression (CHEF-F2A and CMV-F2A) was monitored for 18 weeks in the absence of the selective pressure. Every two weeks, cell expression was assessed by ELISA. A correlation between the expression level and stability was observed. On average, cell pools generated using CHEF-F2A vector maintained about 35% of their initial expression level which was, twice more than those generated using CMV promoter (Fig 6).

### Clonal selection

**Fig 7:**
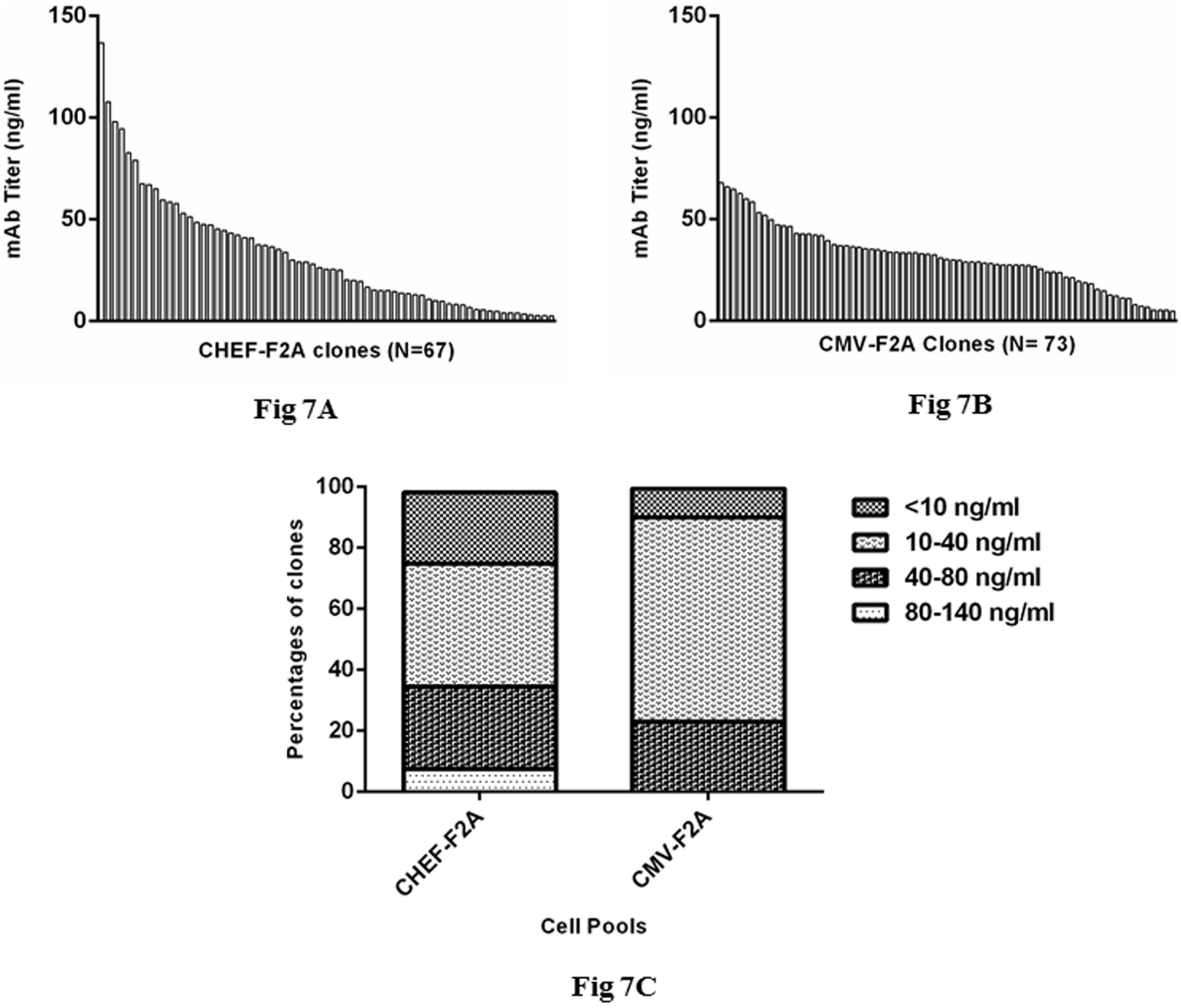
Screening of clonal cell lines established from each stable cell pools transfected with vectors containing F2A sequences. Single cell were generated from CHEF-F2A and CMV-F2A cell pools by limiting dilution technique and expanded for additional analysis. The level of antibody expression was determined by ELISA. (A) Measuring the level of antibody expressed by clonal cell lines derived from CHEF-F2A cell pool. (B) Measuring the level of antibody expressed by clonal cell lines derived from CMV-F2A cell pool. (C) Clonal cell lines recovered from both cell pools were categorized according to level of antibody expression. The percentage of cell lines in each group was indicated.

To evaluate and compare clonal expression of higher producer cell pools, clones were generated from cell pools by limiting dilution. 73 clones per CMV-F2A and 67 clones per CHEF-F2A cell pools were screened for antibody expression to identify the higher producer clones. Clonal expression varied between 5 to 140 ng/ml. Clones derived from the transfection of CHEF-F2A construct showed higher expression level compared to clones derived from CMV-F2A cell pools (Fig 7). As it has been indicated in Fig 7, about 8% of the clones obtained from CHEF-F2A cell pools expressed the mAb at more than 80 ng/ml while none of the clones derived from CMV-F2A cell pools expressed the mAb at this concentration. Of note, 23% of single clones derived from the CHEF-F2A cell pools expressed the mAb lower than 10 ng/ml while about 9% of CMV-F2A single clones exhibited this level of expression.

### Determining copy number

**Fig 8:**
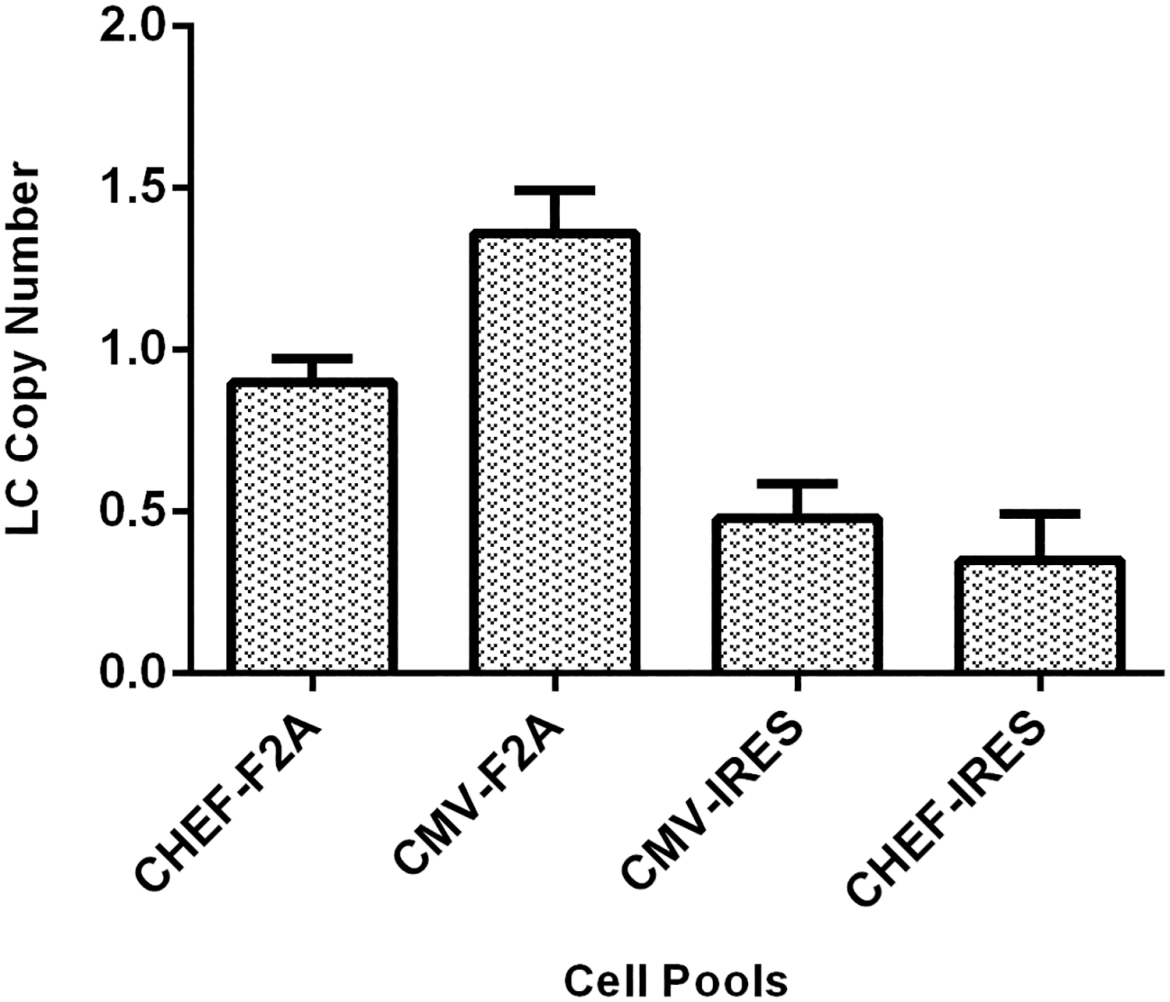
Analysis of integrated transgene copy number in stable cell pools transfected by four bicistronic vectors. Antibody copy number based on light chain copy number was calculated by qRT-PCR. The error bars represent the standard deviation of three independent measurements.

Gene copy number (GCN) of integrated transgene in the host genome was calculated by qRT-PCR. The stable cell pools transfected with either CHEF-F2A or CHEF-IRES vector contained fewer copies of the transgene compared with the cell pools transfected either CMV-F2A or CMV-IRES vectors (Fig 8). Comparing GCN with relative mRNA expression, no correlation was found. Cell pools transfected with CMV-based vectors’ had higher GCN, but lower mRNA expression in comparison to stable cell pools which received the CHEF–based plasmids.

## Discussion

Employing strong promoters is an essential parameter for mAb expression in CHO cells. Although CMV is the most commonly used promoter for recombinant protein production in CHO cells, several studies have described its potential silencing and inactivation [7]. The CHO-derived elongation factor-1 promoter (CHEF-1) has been described as a promising tool for driving high-level expression in mammalian cells compared to other kinds of promoters [9]. CHEF1-based expression vector containing the 4.1 kb upstream and 4.2 kb downstream flanking regions of the CHO-EF1 gene was used to express recombinant proteins in mammalian cells [9]. Although these flanking sequences improve expression considerably, their incorporation into the promoter region leads to an increase in the vector size which could cause difficulties in cells transfection and genomic stability [27]. Formerly, the efficiency of CHEF promoter region excluding flanking regions was examined for expression of enhanced green fluorescent protein, EGFP, in stem cells and CHO-K1 cells. Considering these investigations, CHEF promoter region [10] was employed for expression of recombinant monoclonal antibodies and comparison of its strength with CMV promoter in CHO-S cells. This is the first report on applying CHEF promoter region to drive mAb expression in CHO-S cells.

To study the efficiency of CHEF and CMV promoters for the expression of mAb in stably transfected CHO-S cells, four bicistronic vectors were designed. These constructs comprised either IRES or 2A self-cleavable sequence and expressed mAb under the control of either CMV or CHEF promoters.

Stable cell lines expressing monoclonal antibody were produced using constructed vectors. The results in both mRNA and protein levels showed that stable cell pools containing vectors with CHEF promoter had higher expression level in comparison with cell pools containing CMV promoter. Interestingly, this enhancement was more significant in cell pools expressing antibody via F2A system. Additionally, the CHEF-F2A vector demonstrated 2.5–2.8 times higher expression in comparison to the CMV-F2A promoter. This observation was consistent with another study which reported that the activity of CHEF promoter to be 2.5 to 3.5 fold higher than CMV promoter in both HES-2 and HES-3 cell lines [10]. However, Ho. et al described CHO EF1-a promoter gave lower EGFP expression than CMV promoter in stably transfected CHO K1 cells [28].

While single clones derived from CHEF-F2A and CMV-F2A cell pools were analyzed, expression level of CHEF-F2A single cells was notable. Although the high producer clones were obtained from CHEF-F2A cell pools, the percentage of low producer (< 10ng/ml) was more than CMV-F2A single clones. Growth advantage of low- producer population over high producer population could lead to appearance of more low- producer clonal cell received CHEF promoter [29]. Site of integration of CHEF promoter in CHO genome, which was intrinsically random, might be one factor for heterogene expression of clones. Kaufman and his colleges showed that site of integration could cause heterogenous expression of transgene. They hypothesized that such loci might be favorable for expression of antibiotic resistance gene, but it could not support expression of transgene [30]. However further analyses of clones are needed to address these clonal variations.

Comparing two bicistronic systems, it was revealed that F2A-system was more efficient than IRES based vectors in mAb expression which was in agreement with previous reports [17, 31–35]. CHEF-F2A vector showed the highest expression of other transfectants. These findings suggested that apart from promoters, vector design in terms of linker choices had great impacts on the expression of monoclonal antibody.

Furthermore, no correlation between the plasmid copy number and the expression level of mAb was observed. For example, cells expressing the transgene under the control of CMV promoter received more copies of the plasmid. However they had lower transcription and expression level. The results are consistent with former studies in which GCN did not reflect the content of mRNA [36]. Studies have shown that mammalian cells lines which contain higher copies of the heterologous plasmids were are susceptible to silencing [37, 38], and early methylation coincide with high transgene copy numbers [26]. Since CMV-F2A and CMV-IRES transfected cells carried more copies of transgene than cells receiving CHEF-F2A and CHEF-IRES vectors, their lower expression might be due to gene silencing.

As higher expression level was achieved via F2A constructs, we performed additional experiments to analyze the expression of the mAb in cell pools bearing F2A vectors. First, we evaluated the long-term expression of antibody in these cell pools upon the removal of the selective pressure. After 18 weeks, CHEF promoter harboring cells retained 35% of their mAb expression level while cells having CMV promoter gradually lost their expression, and only 10% of their mAb expression retained. Reduction in the expression of the mAb via CMV promoter over time can be caused by promoter silencing [39, 40] due to DNA methylation [7, 26] or loss of activating histone modifications [41–43].

Second, we analyzed the expressed antibody by western blotting. Western blot analysis of antibody expressed via IRES-mediated vectors showed two distinct bands of 25 kDa and 50 kDa corresponding to the LC and HC, respectively However antibody expressed by F2A system had three distinct bands of 25, 28, and 30 kDa, corresponding to the light chain. It has been proposed that different arrangments of the antibody chains and furin recognition site can influence the performance of furin [31]. However, when we placed the LC gene upsreatm of the furin recognition site, we did not observe complete cleavage of furin. In agreement with our studies, several other investigations have also indicated that furin could not cleave efficiently when either LC or HC were arranged as a first cisteron [31, 44, 45]. Furthermore, different furin cleavage patterns were detected in the cell pools receiving either CHEF-F2A or CMV-F2A vectors. Considering these finding, it appears that factors other than the arrangement of the light and heavy chains affect the hydrolysis activity of furin. To give an illustration, Fang et al. observed complete furin cleavage upon expression of mAb *in vivo* (Rat) [19] while furin could not cleave completely when mAbs were expressed in CHO and HEK 293T cells [31, 44, 45]. Also, some reports have demonstrated that secretory pathway of CHO cells is not able to provide sufficient amount of proteolytic enzymes to support the complete processing of the recombinant proteins. To ensure complete cleavage of furin to obtain antibody chains with correct size, it is suggested to co-express full-length furin in CHO cells [46–49].

In conclusion, we have been able to show that CHEF-F2A system elicits higher expression level and stability compared with other studied constructs and hence it is a viable system for successful expression of antibodies. However, further studies to improve both the level and stability of the expression and efficiency of the furin cleavage are required.
